# Dicer represses the interferon response and the double-stranded RNA-activated protein kinase pathway in mouse embryonic stem cells

**DOI:** 10.1016/j.jbc.2021.100264

**Published:** 2021-01-08

**Authors:** Chandan Gurung, Mona Fendereski, Krishna Sapkota, Jason Guo, Faqing Huang, Yan-Lin Guo

**Affiliations:** 1 Department of Cell and Molecular Biology, The University of Southern Mississippi, Hattiesburg, Mississippi, USA; 2 Department of Chemistry and Biochemistry, The University of Southern Mississippi, Hattiesburg, Mississippi, USA

**Keywords:** embryonic stem cell, Dicer, interferon response, innate immunity, double-stranded RNA-activated protein kinase, cytotoxicity, RNA interference, CM, conditioned medium, ESC, embryonic stem cell, GFP, green fluorescent protein, IFN, interferon, ISG, IFN-stimulated gene, PKR, protein kinase R, RIG-1, retinoic acid–inducible gene I, RT-qPCR, real-time quantitative polymerase chain reaction, SINE, short interspersed nuclear element, TE, transposable element, TLR, toll-like receptor

## Abstract

Recent studies have demonstrated that embryonic stem cells (ESCs) are deficient in expressing type I interferons (IFN), the cytokines that play key roles in antiviral responses. However, the underlying molecular mechanisms and biological implications of this finding are poorly understood. In this study, we developed a synthetic RNA-based assay that can simultaneously assess multiple forms of antiviral responses. Dicer is an enzyme essential for RNA interference (RNAi), which is used as a major antiviral mechanism in invertebrates. RNAi activity is detected in wild-type ESCs but is abolished in Dicer knockout ESCs (D−/−ESCs) as expected. Surprisingly, D−/−ESCs have gained the ability to express IFN, which is otherwise deficient in wild-type ESCs. Furthermore, D−/−ESCs have constitutively active double-stranded RNA (dsRNA)-activated protein kinase (PKR), an enzyme that is also involved in antiviral response. D−/−ESCs show increased sensitivity to the cytotoxicity resulting from RNA transfection. The effects of dsRNA can be partly replicated with a synthetic B2RNA corresponding to the retrotransposon B2 short interspersed nuclear element. B2RNA has secondary structure features of dsRNA and accumulates in D−/−ESCs, suggesting that B2RNA could be a cellular RNA that activates PKR and contributes to the decreased cell proliferation and viability of D−/−ESCs. Treatment of D−/−ESCs with a PKR inhibitor and IFNβ-neutralizing antibodies increased cell proliferation rate and cell viability. Based on these findings, we propose that, in ESCs, Dicer acts as a repressor of antiviral responses and plays a key role in the maintenance of proliferation, viability, and pluripotency of ESCs.

The innate immune system is the first line of an organism’s defense against a broad range of pathogen invasions. Although innate immunity consists of different mechanisms, the antiviral response is one of the most critical components and is presumably developed in most, if not all, mammalian cells (1, 2). However, a series of our recent studies have demonstrated that mouse embryonic stem cells (ESCs) have an attenuated innate immune response. In particular, they do not express type I interferons (IFN) and lack response to inflammatory cytokines. Similar observations have been made in human ESCs and induced PSCs (iPSCs) (3, 4). Therefore, this is a common property shared by all types of PSCs. It appears that ESCs in the early embryo are immunologically divergent from the traditional view of “innate immunity” established in somatic cells of developed organisms.

The biological implications of the attenuated innate immune responses in ESCs have been speculated from different perspectives. Immune response is a double-edged sword: it serves as a critical part of the defense mechanism, but it can also cause immunologic toxicity to tissues since IFN and inflammatory cytokines negatively impact cell proliferation and viability (5, 6, 7). While this could be tolerated by tissues of developed organisms, it could cause serious damage to ESCs in an early embryo. From this perspective, an attenuated immunological response could serve as a self-protective mechanism in ESCs by minimizing immunological cytotoxicity at early stages of embryogenesis (8). From the perspective of ESC biology, IFN response does not appear to be compatible with the pluripotency of ESCs, as demonstrated by a recent study showing that forced activation of the IFN pathway can cause dysregulation of many pluripotency- and lineage-specific genes in ESCs (9). Both scenarios are supported by strong experimental evidence and are not mutually exclusive. However, they only make biological sense if the deficiency in IFN production does not compromise the defense capacity of ESCs. Indeed, two alternative antiviral mechanisms have been proposed; ESCs may use a subset of preexisting IFN-stimulated genes (ISGs) that are independent of IFN stimulation (10), or they may use the RNA interference (RNAi) antiviral pathway that may not be operational in differentiated mammalian cells (11).

Although RNAi is widely recognized as a major antiviral mechanism in invertebrates, such a function has not been convincingly demonstrated in mammals. Interestingly, RNAi activity was detected in viral infected mouse ESCs, pointing to the possibility that RNAi could be an alternative antiviral mechanism in ESCs, in which the IFN system is deficient (12). However, the physiological significance of RNAi as an antiviral mechanism in ESCs remains uncertain (13). Since Dicer is the key enzyme responsible for miRNA and siRNA biogenesis, D−/−ESCs could be used as a loss-of-function model to investigate the function of RNAi since they have retained the basic morphology of ESCs and the capacity to express pluripotency markers even though they display severe differentiation and growth defects (14, 15). Interestingly, it was recently reported that D−/−ESCs were able to express IFNβ and show increased antiviral activity (16). Quite unexpectedly, our studies using ESCs as a model to express proteins from synthetic mRNA have led to new insights into the role of Dicer in the regulation of antiviral responses in these cells. Direct expression of a protein from its synthetic mRNA is an alternative to plasmid DNA- or viral vector-based gene expression systems (17). A major biological issue with this method is that synthetic mRNA transfected to the host cells is detected as foreign RNA and elicits antiviral responses, leading to reduced cell viability and apoptosis of the host cells (17). However, this is not a serious problem in ESCs due to their attenuated antiviral responses as we demonstrated in a recent study (18). Taking advantage of this feature in ESCs, we attempted to use green fluorescent protein (GFP) expressed from its synthetic mRNA as a virus-free *in vitro* assay to determine RNAi activity in this study. RNAi activity was indeed detected in wild-type ESCs, but not in D−/−ESCs, as expected. However, D−/−ESCs showed increased antiviral responses to RNA transfection.

Viral RNA induces IFN response by interacting with toll-like receptors (TLRs) and retinoic acid–inducible gene I (RIG-I)-like receptors (RLRs), leading to IFN transcription through activation of NFκB and IRFs (19, 20, 21). In addition, viral RNA can also activate other antiviral mechanisms, such as dsRNA-activated protein kinase R (PKR). Activation of PKR causes inhibition of both cellular and viral protein synthesis. While this represses viral replication, it also inhibits cell proliferation (22). PKR is constitutively expressed in cells and is readily activated by viral dsRNA or by dysregulated cellular RNA, but it can be further upregulated by IFN as a part of the IFN response. Thus, the IFN system can activate multiple pathways and mount a powerful antiviral response (7).

While it is apparent that the deficiency of ESCs in expressing IFN is closely related to pluripotency, the underlying molecular basis of this deficiency is poorly understood. In this study, we demonstrate that D−/−ESCs have gained the ability to express not only the type I IFN system, but also constitutively active PKR, which together contribute to the reduced cell proliferation and cell viability of D−/−ESCs. Our data revealed a critical role of Dicer as a repressor of antiviral responses in ESCs, which represents a novel mechanism essential for ESCs to maintain rapid proliferation and to prevent potential cell damage resulting from dysregulated endogenous RNA transcripts.

## Results

### Development of an RNA-based assay to determine different antiviral responses in ESCs

This assay was initially intended to determine the RNAi activity in ESCs. Functionalized GFP-mRNA was first transfected into cells where it was translated to GFP. The cells were then transfected with a synthetic dsRNA corresponding to the sequence of GFP (designated as dsGFP). Based on the principle of RNAi, dsGFP would be processed to yield siRNA that will specifically target GFP-mRNA, thereby reducing GFP expression. GFP was detected as early as 3 h after GFP-mRNA transfection. Subsequent transfection with either dsGFP or dsLuc (a control luciferase dsRNA of similar length with a sequence unrelated to GFP-mRNA) reduced GFP expression as indicated by diminished green fluorescence (Fig.S1*A*). Quantitative analysis by flow cytometry indicated that GFP fluorescence intensity in ESCs was reduced more by dsGFP than by dsLuc (Fig.S1*B*, 59% *versus* 76% at 24 h, in comparison with control, 100%). However, in D−/−ESCs, both dsGFP and dsLuc reduced the expression of GFP to a similar level (45% *versus* 47%, at 24 h). A logical explanation for these results would be that the different effect between dsGFP and dsLuc in ESCs is due to sequence-specific reduction of GFP-mRNA by dsGFP *via* Dicer-dependent RNAi activity, which is abolished in D−/−ESCs. The non-sequence-specific effects in the reduction of GFP fluorescence caused by dsGFP and dsLuc in both ESCs and D−/−ESCs are likely due to the activation of other pathways.

These results demonstrated the existence of RNAi activity in ESCs, which is in agreement with the study using GFP expressed from plasmids as an siRNA target (23). However, the most notable observation is that transfection of D−/−ESCs with dsRNA, and to a lesser extent with GFP-mRNA, caused cell death, as judged by the increased number of detached cells (Fig.S1*A*, D−/−ESC1), whereas the cytotoxicity caused by dsRNA transfection in ESCs is low and limited to reduced colony size (Fig.S1*A*, ESC1). These results suggested that D−/−ESCs have increased susceptibility to the cytotoxicity of transfected RNA. The GFP-mRNA contains some uncapped GFP-mRNA, which has a 5’ppp-group that can activate RIG-I (24). Therefore, the RNA preparations used in the above experiments can potentially interact with most, if not all, known RNA receptors, including TLR3, RIG-I, MDA5, and PKR, that can potentially activate most of the common antiviral pathways. Therefore, cellular effects of RNA transfection on ESCs and D−/−ESCs represent the collective results of their antiviral responses. The increased antiviral response in D−/−ESCs is a novel finding that we investigated further with two independent pairs of ESC lines (ESC1;D−/−ESC1 and ESC2;D−/−ESC2) in this study.

### D−/−ESCs have increased capacity to express type I IFN

**Figure 1:**
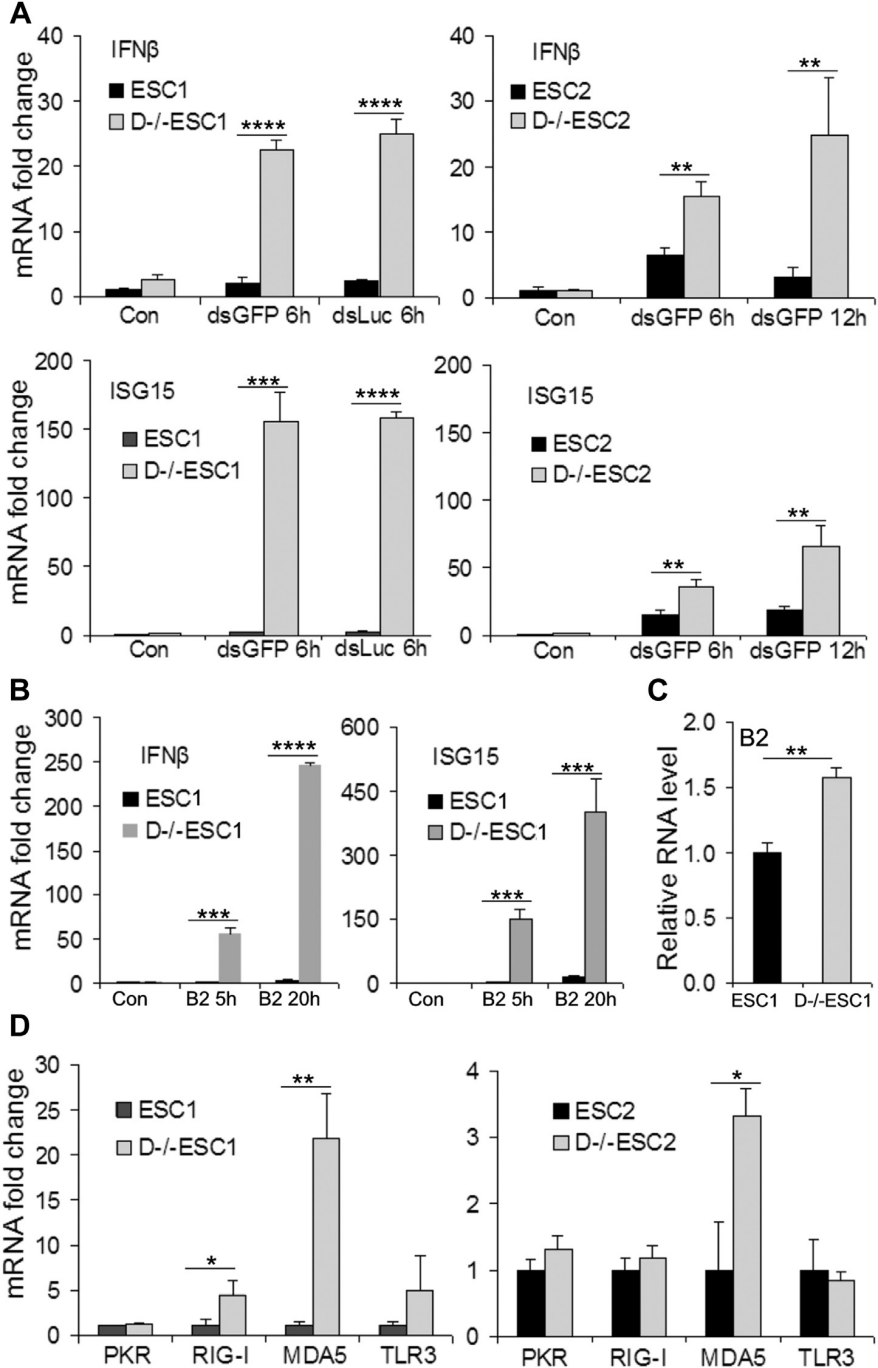
**dsRNA- and B2RNA-induced expression of IFNβ and ISG15 in ESCs and D−/−ESCs.** ESCs and D−/−ESCs were transfected with dsRNA or B2RNA for the indicated time periods. The mRNA levels of the tested genes were determined by RT-qPCR. *A*, dsGFP- and dsLuc-induced IFNβ and ISG15 mRNA. *B*, B2RNA-induced IFNβ and ISG15 mRNA. *C*, relative cellular levels of B2RNA in ESCs and D−/−ESCs. *D*, Comparison of the basal mRNA levels of dsRNA receptors in ESCs and D−/−ESCs. For *A* and *B*, the mRNA level of each tested gene in the control ESCs (Con) is designated as 1. For *C* and *D*, the basal mRNA level of each tested gene in ESCs is designated as 1. The values are as mean ± SD of three independent experiments (*A* and *D*) or a representative experiment performed in biological triplicate that was performed at least twice (*B* and *C*). *p* < 0.0001,∗∗∗∗; *p* < 0.001,∗∗∗; *p* < 0.01,∗∗; *p* < 0.05.∗ Compared groups are indicated by a horizontal bar.

To determine the effect of Dicer deletion on the IFN pathway, we transfected the cells with dsGFP or dsLuc and analyzed the expression of IFNβ and ISG15 as indicators of the functionality of the type I IFN system. As shown in Figure 1*A*, the mRNA levels of the two genes were not or only slightly increased in ESCs, but they were strongly stimulated in D−/−ESCs. Similar results were observed when cells were transfected with polyIC (polyinosinic-polycytidylic acid), a synthetic dsRNA used as a viral RNA analog in our previous studies (25, 26) (data not shown).

It is known that certain cellular RNA with dsRNA structures, including those from apoptotic cells, misprocessed RNA, and transcripts of transposable elements (TEs), can induce antiviral responses and cause cellular damage in the absence of infection (27, 28, 29). The B2 short interspersed nuclear element (SINE) is a major type of retrotransposons in mouse genomes (30). B2RNA, which has several features of dsRNA (31), is abundantly expressed in mouse ESCs. As shown in Figure 1*B*, synthetic B2RNA (B2) showed patterns similar to dsGFP and dsLuc in inducing IFNβ and ISG15 expression in ESCs and D−/−ESCs. It is noted that the endogenous B2RNA level in D−/−ESCs was significantly higher than in ESCs, indicating its accumulation in D−/−ESCs (Fig. 1*C*). If Dicer is responsible for processing endogenous B2RNA, it could target the transfected B2 RNA as well. To test this possibility, we transfected ESCs and D−/−ESCs with Cy3-labeled fluorescent B2RNA (Cy3-B2) and analyzed the levels of Cy3-B2 by Cy3 fluorescence intensity. Our results indicated that Cy3-B2 was detected at higher levels in D−/−ESCs than in ESCs (Fig. S2), consistent with the accumulation of endogenous B2RNA in D−/−ESCs (Fig. 1*C*). We also compared the basal mRNA levels of the RNA receptors that mediate the effects of viral RNA. With the exception of MDA5, which is expressed at higher levels in D−/−ESCs than in ESCs, the other tested dsRNA receptors are expressed at comparable levels (Fig. 1*D*).

### D−/−ESCs have a functional type I IFN system

**Figure 2:**
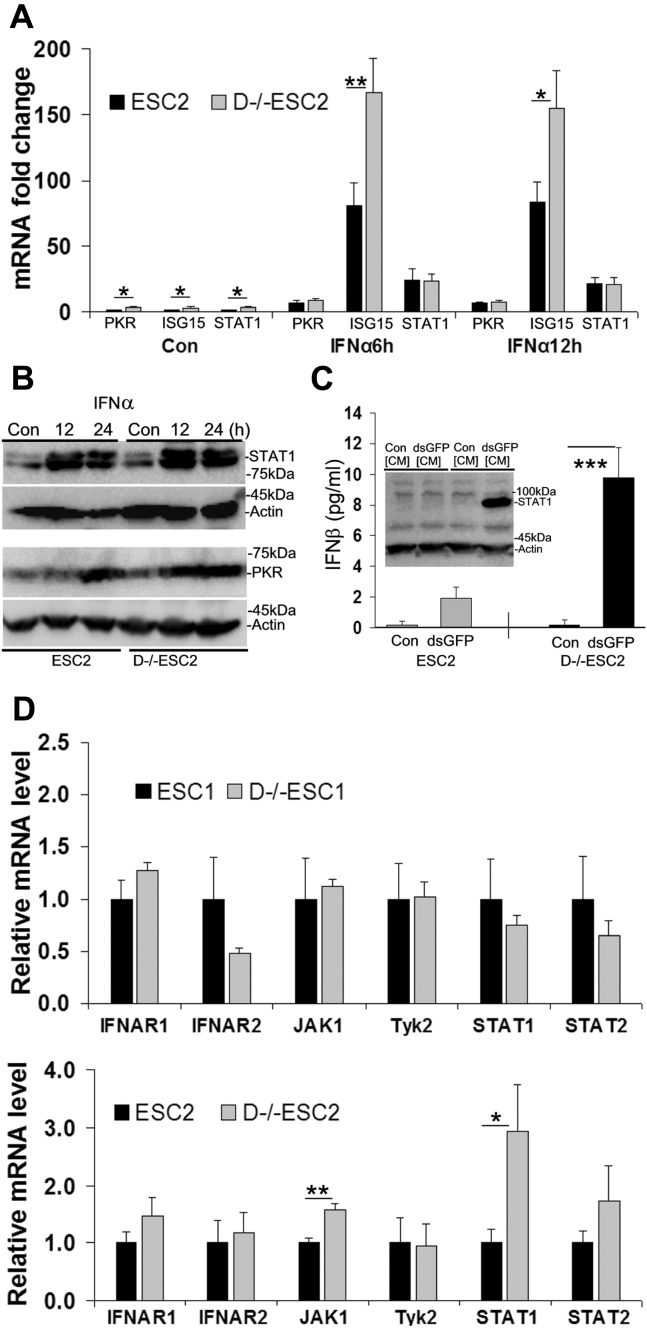
**IFN responses in ESCs and D−/−ESCs.***A*, RT-qPCR analysis of IFNα (500 units/ml)-induced PKR, ISG15, and STAT1 expression. The mRNA level of each tested gene in the control ESCs (Con) is designated as 1. *B*, western blot analysis of IFNα-induced expression of STAT1 and PKR. *C*, blot inset, conditioned medium (CM)-induced expression of STAT1. ESC-FBs were treated for 24 h with Con[CM] (prepared from ESCs and D−/−ESCs without dsGFP transfection) and dsGFP[CM] (prepared from ESCs and D−/−ESCs transfected with dsGFP). Con, cells without any treatment. Bar graph, ELISA analysis of IFNβ in the medium secreted by ESCs and D−/−ESCs in response to dsGFP. *D*, RT-qPCR analysis of the basal mRNA levels of signaling molecules that mediate the effects of type I IFN in ESCs and D−/−ESCs. The basal mRNA level of each tested gene in ESCs is designated as 1. In bar graphs, the values are as mean ± SD of three independent experiments (*C* and *D*) or a representative experiment performed in biological triplicate that was performed at least twice (*A*). *p* < 0.0001,∗∗∗∗; *p* < 0.001,∗∗∗; *p* < 0.01,∗∗; *p* < 0.05.∗ Compared groups are indicated by a horizontal bar. In Western blot analysis, the blots are representatives from experiments that were repeated three times. β-Actin was used as a reference for protein loading.

ESCs are able to respond to IFNα and IFNβ, but they are unable to express the two cytokines (25, 26). To test if Dicer deletion affects the responsiveness of ESCs to IFN, we treated the cells with IFNα and compared IFNα-induced expression of three ISGs (PKR, ISG15, and STAT1) in ESCs and D−/−ESCs. IFNα induced expression of all three genes, with ISG15 mRNA induced significantly higher in D−/−ESCs than in ESCs (Fig. 2*A*). It is also noted that the basal mRNA levels of all three ISGs are about threefold higher in D−/−ESCs than in ESCs. At the protein level, STAT1 was detected in unstimulated cells, but it was induced by IFNα in both ESCs and D−/−ESCs. IFNα-induced PKR was also apparent in ESCs and D−/−ESCs (Fig. 2*B*, left panel).

For a functional analysis of the IFN system, we prepared conditioned medium (CM) from dsGFP-transfected ESCs and D−/−ESCs (dsGFP[CM]). The rationale is that if the CM contains IFN, it will induce ISGs, such as STAT1, resembling the effects of IFNα (Fig. 2*B*). In this experiment, we used ESC-differentiated fibroblasts (ESC-FBs) since they are much more responsive to IFN than ESCs (32). As shown in Figure 2*C*, dsGFP[CM] prepared from D−/−ESCs, but not that from ESCs (blot inset), induced the expression of STAT1 in ESC-FBs. This result indicated that dsGFP[CM] only from D−/−ESCs contains secreted IFN, as confirmed by ELISA analysis of IFNβ in the CMs (Fig. 2*C*, bar graph). Together, these data demonstrate that D−/−ESCs have a fully functional type I IFN system.

We further analyzed the expression levels of the major signaling molecules that mediate the effects of IFN. As shown in Figure 2*D*, RT-qPCR analysis indicated that most of these molecules were expressed at comparable mRNA levels in ESCs and D−/−ESCs although some variations were noted for JAK1, IFNAR2, and STAT1 between the two different D−/−ESC lines.

### The PKR pathway is constitutively active in D−/−ESCs

**Figure 3:**
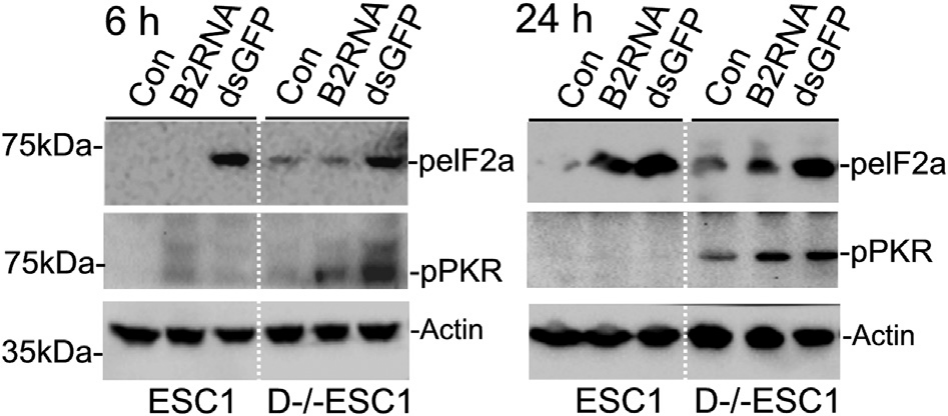
**PKR activation in ESCs and D−/−ESCs**. Cells were transfected with dsGFP or B2 RNA for 6 h and 24 h. PKR activation was determined by the levels of phosphorylated eIF2α (peIF2α, a substrate of PKR) and phosphorylated (activated) PKR (pPKR) with Western blot. β-Actin was used as a reference for protein loading. The blots are representatives from experiments that were repeated three times.

The PKR pathway is functional in ESCs as we demonstrated with polyIC transfection and viral infection (25). To determine if this pathway is also altered in D−/−ESCs, we treated the cells with dsGFP or B2RNA. Although dsRNA is the best characterized activator of PKR, single-stranded RNA having hairpin structures with a certain length can also activate PKR (27, 28, 29). Therefore, we speculated that B2RNA may be able to activate PKR since it has a secondary structure with five hairpins, two loops, and two single-stranded regions (31). PKR activation is commonly assessed by the phosphorylation of eukaryotic initiation factor 2α (peIF2α), which is a well-characterized PKR substrate (6), and by the level of phosphorylated PKR (pPKR), which is the active form of PKR. As shown in Figure 3, peIF2α was detected in both ESCs and D−/−ESCs treated with dsGFP and B2RNA, but levels of pPKR were much higher in D−/−ESCs than in ESCs. However, the most notable observation is that both pPKR and peIF2α were detected in untreated D−/−ESCs but barely detected in untreated ESCs (Fig. 3 Con), indicating that PKR is constitutively active in D−/−ESCs.

### D−/−ESCs have reduced cell proliferation rate and express high levels of cell cycle inhibitors

**Figure 4:**
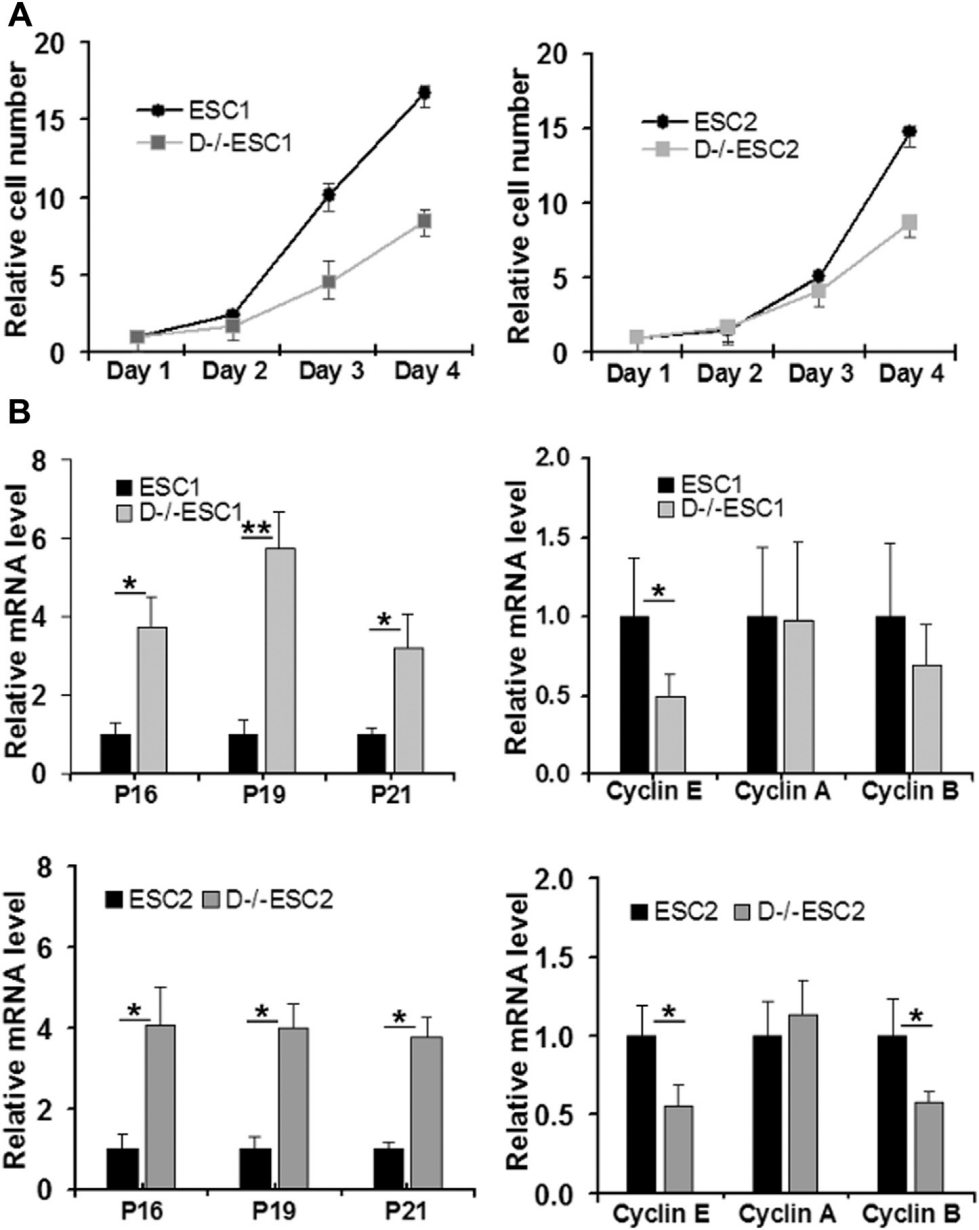
**Analysis of cell proliferation and cell cycle regulator expression in ESCs and D−/−ESCs.***A*, cell proliferation analysis. Cells were seeded at about 30% confluence in a 48-well plate. The cell numbers at the indicated time points were determined by cell viability assay. The cell number at day 1 for both ESCs and D−/−ESCs is set as 1. *B*, RT-qPCR analysis of mRNA levels of cell cycle regulators. The basal mRNA level of each tested gene in ESCs is designated as 1. The values are as mean ± SD of three independent experiments (*B*) or a representative experiment performed in biological triplicate that was performed at least twice (*A*). *p* < 0.0001,∗∗∗∗; *p* < 0.001,∗∗∗; *p* < 0.01,∗∗; *p* < 0.05.∗ Compared groups are indicated by a horizontal bar.

ESCs are characterized by their rapid cell proliferation rate due to constitutively activated cyclin-dependent kinases (CDKs) driven by high expression levels of cyclins A, B, and E (33). D−/−ESCs have a substantially lower growth rate than ESCs (Fig. 4*A*). RT-qPCR analysis indicated that the mRNA level of cyclin E and cyclin B appeared to be lower in D−/−ESCs than in ESCs while cyclin A was not significantly altered. However, three major cell cycle inhibitors, p21, p19, and p16, which are expressed at very low levels in ESCs, are expressed at substantially higher levels in D−/−ESCs (Fig. 4*B*). These results explain, at least partially, the reduced cell proliferation in D−/−ESCs.

### Contribution of PKR activation to the reduced cell proliferation in D−/−ESCs

**Figure 5:**
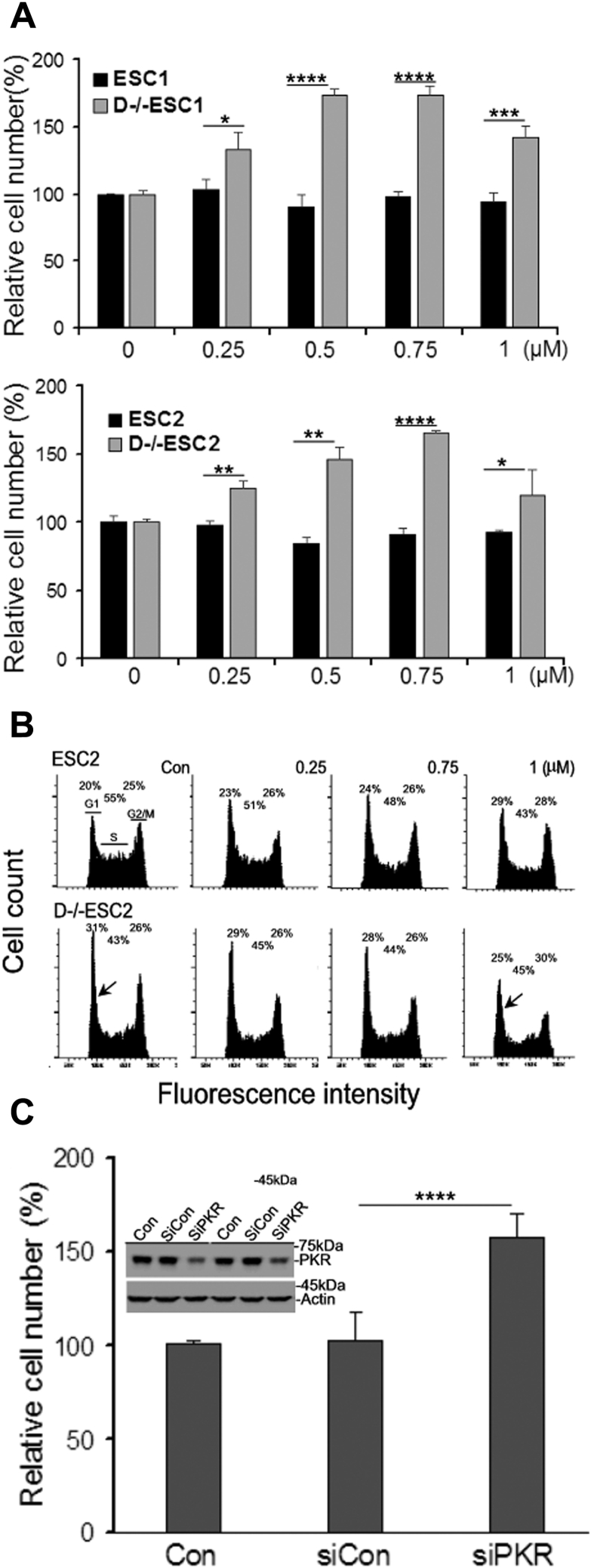
**Effects of PKR inhibition on cell proliferation and cell cycle of ESCs and D−/−ESCs.***A*, cells were seeded at about 30% confluence and cultured in the absence (Con) or presence of PKR inhibitor C16 at the indicated concentrations. The cell numbers were determined by cell viability assay after 60 h incubation. The cell number in the control (Con) was set as 100%. *B*, cells were treated under the conditions as described in *A* for 48 h. Cell cycle was analyzed by flow cytometry. *Insets* show percentages of cell populations in different phases. *Arrows* denote the changes of G1 phase cells in control and C16 (1 μM)-treated cells. The histograms are representatives of flow profiles from experiments that were repeated three times with similar results. *C*, cells were transfected with siRNA against PKR (siPKR) or control siRNA (siCon). After 48 h incubation, the cell number was determined by the method described in *A*. The number of cells without transfection (Con) was set as 100%. PKR knockdown is assessed by Western blot analysis. The blot inset shows two sets of independent samples. In bar graphs, the values are as mean ± SD of a representative experiment performed in biological triplicate that was performed at least twice (*A*) or the mean ± SD of two combined independent experiments each performed in biological triplicate (*C*). *p* < 0.0001,∗∗∗∗; *p* < 0.001,∗∗∗; *p* < 0.01,∗∗; *p* < 0.05.∗ Compared groups are indicated by a horizontal bar.

PKR activation is known to inhibit cell proliferation, including in ESCs as we previously demonstrated with polyIC-activated PKR (25). To test if constitutively activated PKR in D−/−ESCs contributed to their reduced cell proliferation, we treated ESCs and D−/−ESCs with an imidazolo-oxindole PKR inhibitor (C16), which shows a similar effect to siRNA that knocks down PKR (25). As shown in Figure 5*A*, C16 treatment significantly increased the cell number of D−/−ESCs in a dose-dependent manner with the maximal effect at the concentration of 0.75 μM, but this dose dependence was not seen in ESCs. We further analyzed the cells by flow cytometry. ESCs have large populations at the S phase and similar cell populations at the G1 and G2 phases when they are grown at low density. However, in comparison, D−/−ESCs have increased G1 cell populations and reduced S phase cell populations, which are indicators of slowed cell cycle progression. Treatment of D−/−ESCs with C16 reduced the G1 cell population. At 0.75 μM and 1 μM, C16-treated D−/−ESCs have a cell cycle profile with a ratio of G1 and G2 cells similar to ESCs. Although the S phase cell population in ESCs was slightly reduced by C16, the overall cell cycle profiles were not altered (Fig. 5*B*), consistent with the unchanged cell proliferation rate (Fig. 5*A*). The effect of C16 on the inhibition of PKR was confirmed by knocking down PKR expression with its specific siRNA, leading to increased cell proliferation similar to the effect of C16 (Fig. 5*C*).

**Figure 6:**
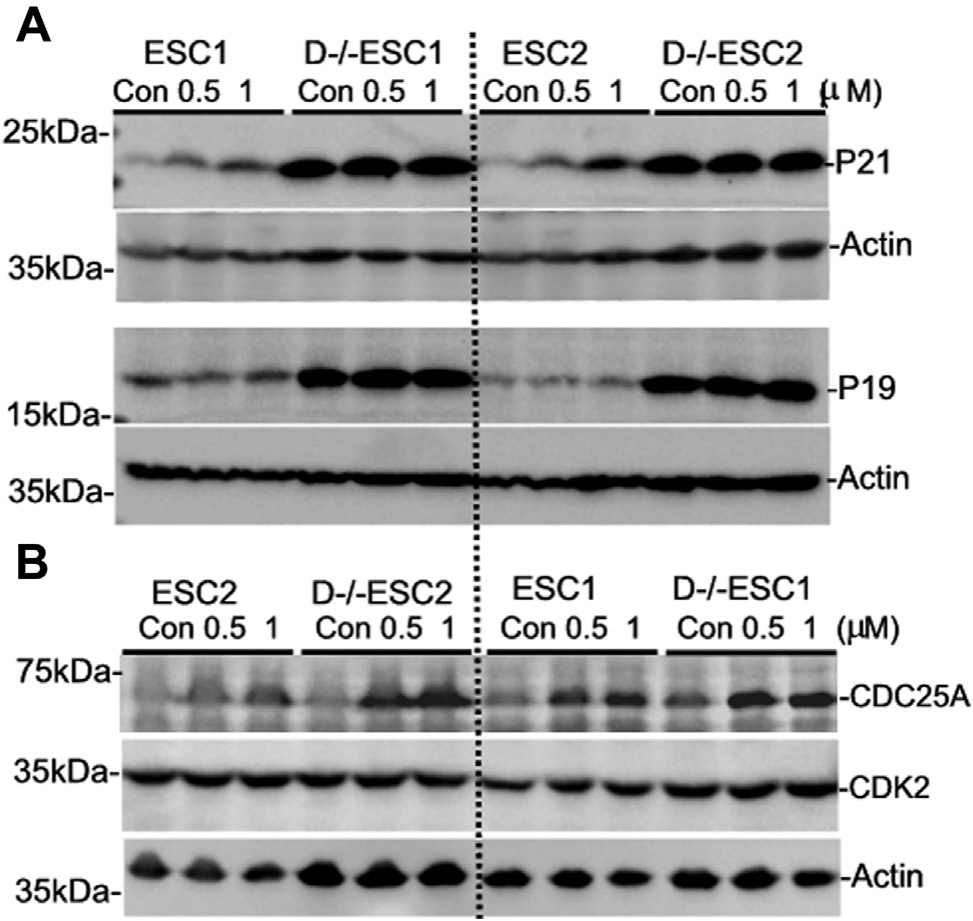
**Effects of PKR inhibition on the expression of cell cycle regulators.** Cells were treated with different concentrations of C16 for 24 h and analyzed by Western blot. β-Actin was used as a reference for protein loading. The blots are representatives from experiments that were repeated three times with similar results.

In line with the results from RT-qPCR analysis shown in Figure 4*B*, western blot analysis indicated that p21 and p19 proteins were expressed at substantially higher levels in D−/−ESCs than in ESCs, but their relative levels were not affected by C16 (Fig. 6*A*). The relative levels of CDK2 (Fig. 6*B*) and cyclin E and A were not significantly altered in either D−/−ESCs or ESCs by C16 treatment (data not shown). However, CDC25A, a protein phosphatase that activates CDKs and leads to G1/S cell cycle progression (34), is upregulated in D−/−ESCs (Fig. 6*B*). This result could contribute to the reduction of G1 phase cells by C16 in D−/−ESCs.

### The contributions of PKR and IFNβ to the cytotoxicity of transfected RNA in D−/−ESCs

**Figure 7:**
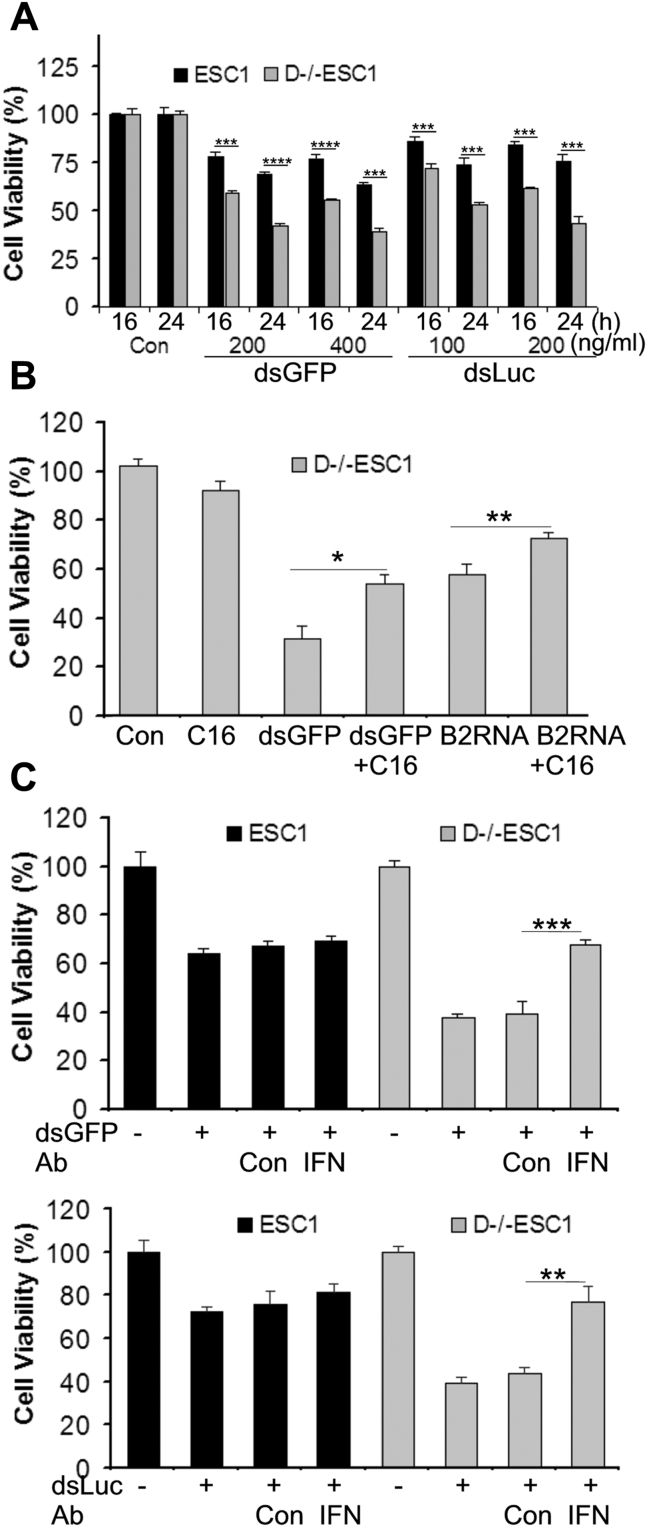
**Contributions of PKR activation and IFN response to the cytotoxicity caused by RNA transfection in ESCs and D−/−ESCs.***A*, effects of dsGFP and dsLuc transfection on cell viability. *B*, inhibition of PKR reduced cytotoxicity caused by dsGFP and B2 RNA transfection in D−/−ESCs. Cells were pretreated with C16 (1 μM) for 30 min followed by transfection with dsGFP or B2 RNA. The cell viability was determined after 24 h incubation. *C*, IFNβ-neutralizing antibodies reduced cytotoxicity caused by dsGFP and dsLuc transfection in ESCs and D−/−ESCs. Cells were preincubated with control isotype antibodies (Ab, Con) or IFNβ-neutralizing antibodies (Ab, IFN) (2 μg/ml) for 30 min followed by transfection with dsGFP or dsLuc. The cell viability was determined after 24 h incubation. The cell number in the control (Con, cells without any treatment) was set as 100%. In all experiments, the values are as mean ± SD of a representative experiments performed in biological triplicate that were performed at least twice. *p* < 0.0001,∗∗∗∗; *p* < 0.001,∗∗∗; *p* < 0.01,∗∗; *p* < 0.05∗. Compared groups are indicated by a horizontal bar.

In routine cell culture, it is notable that there are more cells undergoing spontaneous cell death in D−/−ESCs than in ESCs. In particular, D−/−ESCs are more susceptible than ESCs to the cytotoxicity of RNA transfection as shown in Fig. S1*A*. By quantitatively analyzing the number of viable cells after transfection with different concentrations of dsRNA, our results demonstrated that D−/−ESCs are more sensitive to the cytotoxicity of dsGFP and dsLuc at all concentrations tested (Fig. 7*A*). To determine the contribution of PKR to the cytotoxicity caused by RNA transfection, we pretreated cells with C16 to block PKR activation prior to RNA transfection. As shown in Figure 6*B*, the cytotoxicity caused by both dsGFP and B2RNA can be partially reversed by C16 treatment.

We have previously reported that IFNα or IFNβ alone does not have detectable effects on proliferation and viability of ESCs (25, 26) or D−/−ESCs under normal conditions (data not shown). To determine the contribution of IFNβ to the cytotoxicity in the cells transfected with RNA, we first preincubated ESCs and D−/−ESCs with IFNβ-neutralizing antibodies, followed by cell transfection with dsRNA. The rationale of this experiment is that the activity of IFNβ secreted by the cells will be neutralized by the antibodies before its autocrine signaling action. As shown in Figure 7*C*, neither IFNβ-neutralizing antibodies nor control antibodies affect the effect of dsRNA on ESC viability. However, IFNβ-neutralizing antibodies, but not control antibodies, significantly reduced the cytotoxicity of dsGFP and dsLuc on D−/−ESCs (Fig. 7*C*). Similar results were observed when the cells were transfected with polyIC or B2RNA (data not shown). These results suggested that IFNβ contributes to the cytotoxicity only in D−/−ESCs in which it can be produced, but not in ESCs that are deficient in expressing this cytokine. Together, these data suggest that both PKR activation and IFNβ production contribute to the cytotoxicity associated with antiviral responses in D−/−ESCs.

## Discussion

Using GFP expressed from its synthetic mRNA as an siRNA target, we detected Dicer-dependent RNAi activity in ESCs, a conclusion similar to the results reported in a study using GFP expressed from a plasmid (23). However, the features of synthetic RNA as viral RNA analogs allowed us to reveal strikingly different antiviral responses between ESCs and D−/−ESCs. Namely, Dicer deficiency leads to the acquisition of the ability to express type I IFN and constitutive PKR activation in D−/−ESCs. These two features potentially make D−/−ESCs more susceptible to the cytotoxicity associated with antiviral responses, either from viral RNA or from misprocessed cellular RNA. These findings not only provide important insights into the molecular basis underlying the phenotypes of D−/−ESCs, but also reveal the biological function of Dicer in the regulation of ESC immunological properties, pluripotency, and proliferation.

Since the deficiency in expressing type I IFN is a common feature of all types of pluripotent cells, it appears that this is an intrinsic feature inherently related to the pluripotent state (3). However, findings from this study further suggest that lack of IFN expression in ESCs is not entirely restricted by pluripotency, but it is also repressed by Dicer since D−/−ESCs have retained ESC morphology and pluripotency marker expression and in fact fail to exit the pluripotent state (14, 15). These features presumably made D−/−ESCs a seemingly useful model to study RNAi in mammalian cells in the pluripotent state with an underdeveloped IFN antiviral system. However, it is quite surprising to find that D−/−ESCs have gained the ability to express type I IFN as we demonstrated in this study and in a recent report in which increased activity against viral infection was also noted in D−/−ESCs by other investigators (16). This finding renders D−/−ESCs as an undesirable model system to study RNAi antiviral activity in ESCs as we previously intended, and the constitutive activation of PKR in these cells reveals a novel role of Dicer in controlling antiviral responses caused by dysregulated cellular RNA.

The constitutively activated PKR in D−/−ESCs may have several biological implications. The PKR pathway is functional in ESCs and is activated during mitosis by cellular dsRNA in a highly regulated manner (35). We previously reported that polyIC-activated PKR inhibits ESC proliferation, which can be partly reversed by PKR knockdown with siRNA or by C16 PKR inhibitor treatment (25). The effect of polyIC was replicated with synthetic dsRNA and B2RNA in this study. In addition to viral RNA, PKR can be activated by cellular RNA with certain features of dsRNA, such as misprocessed RNA and transcripts of transposable elements (TEs) (27, 28, 29, 36). Dicer is a key component in the pathway for siRNA and miRNA biogenesis. It also plays a critical role in preventing the “sterile inflammatory response” by silencing/processing endogenous RNA, in particular TE transcripts that are especially abundant and active in early embryos (37). In human cells, Dicer deficiency leads to accumulation of Alu, increased antiviral response, and accelerated apoptosis (27, 38, 39). Furthermore, PKR is activated by ectopically expressed Alu (35). B2RNA is the mouse counterpart of Alu in humans (30). B2RNA, accumulated in D−/−ESCs, could be one of many cellular RNAs accumulating from Dicer deletion that contribute to the constitutive activation of PKR, leading to retarded cell cycle progression and reduced cell viability in D−/−ESCs.

While the molecular mechanisms underlying how D−/−ESCs have gained the ability to produce type I IFN remain to be determined, we can logically assume that deficiency in miRNA biogenesis would be a primary reason. In particular, ESCs express a distinct set of miRNA, known as ESC-specific miRNA (ESC-miRNA), that are critical for the maintenance of the stem cell state (40). Although we have rather limited knowledge about the miRNA that specifically controls the innate immunity of ESCs, the most relevant findings are that two members of the miR-290 cluster of ESC-miRNA, miR-291b-5p, and miR-293, directly target the mRNA of the RelA subunit of NFκB (41). This could contribute to the inactive state of NFκB in ESCs since NFκB and IRFs are the key transcription factors that control IFN expression (42). Indeed, both NFκB and IRF3 can be activated in D−/−ESCs by polyIC, but not in ESCs (16). Furthermore, mitochondrial antiviral-signaling protein (MAVS), a signaling molecule that regulates IFN expression, was identified as a target of miR-673 in ESCs as reported in a recent study (16).

The rapid cell proliferation rate of ESCs is mainly driven by high levels of cyclins A and E and the low levels of cell cycle inhibitors (33). ESC-miR-291a-3p, ESC-miR-294, and ESC-miR-295 directly target the mRNA of several molecules, including p21, that inhibit cyclin/CDK activity (43), and this logically explains the high levels of p21 and p19 in D−/−ESCs and their slow rate of proliferation. It appears that PKR inhibition by C16 in D−/−ESCs did not directly affect the expression of p21, p19, or CDK2. However, this treatment increased the expression level of CDC25A, a protein phosphatase that activates CDKs and leads to G1/S cell cycle progression (34), which explains the increased cell proliferation of D−/−ESCs in the presence of C16. It should be pointed out that a comprehensive assessment of PKR activity on the functions of cell cycle regulators is very difficult due to their large numbers and dynamic nature during the cell cycle progression. However, it is fairly certain that global translation inhibition caused by PKR activation *via* phosphorylation of eIF2α (6) could be a major mechanism of action of dsRNA and B2RNA. Likewise, defining the precise contributions of IFN responses to the phenotype of D−/−ESCs is also challenging due to the fact that IFN can exert their effects in numerous ways. Nonetheless, the results from the experiments with IFNβ-neutralizing antibodies demonstrate that IFNβ (and likely the other members of type I IFN) can potentiate the cytotoxic effect of dsRNA, which could, at least partly, act through the induction of *de novo* synthesis of PKR or other ISGs.

The observation that D−/−ESCs can express type I IFN is quite noticeable since normal ESCs do not produce these cytokines. We are not aware of studies that specifically analyze the effect of Dicer knockout on IFN response in differentiated somatic cells. However, it has been reported that Dicer knockdown with siRNA in endometrial cancer cells resulted in an increased IFN response (44). It is also noted that both Dicer knockout mouse embryonic fibroblasts and HEK293 cells show reduced cell proliferation rate (45, 46). These findings suggest that Dicer may share similar functions in ESCs and differentiated cells. However, Dicer may play unique and prominent roles in ESCs in which preventing antiviral responses and maintaining a rapid rate of cell proliferation are fundamentally important for the normal growth and development of an early embryo.

In summary, the data presented in this study further support the hypothesis that antiviral responses could negatively impact ESC function. We have identified Dicer as a repressor of both the IFN system and the PKR pathway in ESCs at the pluripotent state. However, it should be pointed out that Dicer regulates numerous cellular processes, directly or indirectly. The specific mechanisms that lead to the acquisition of the ability to express type I IFN and constitutive PKR activation in D−/−ESCs remain to be determined.

## Experimental procedures

### Cells and cell culture

The immunological properties of mouse ESCs have been investigated with two independent cell lines (D3 and DBA252) in our previous studies (25, 26). Two pairs of wild-type mouse ESCs (ESCs) and Dicer knockout ESCs (D−/−ESCs) were used for most experiments to validate the results. They were designated as ESC1 (D3 cell line, ATCC) and D−/−ESC1 (kindly provided by Dr Gregory Hannon) (14) and ESC2 and D−/−ESC2 (kindly provided by Dr Phillip Sharp) (47). All ESCs and D−/−ESCs were maintained in standard mouse ESC medium that contains leukemia inhibitory factor (LIF) as previously described (48). ESC-differentiated FBs (ESC-FBs, differentiated from D3 ESCs) were cultured in ESC medium in the absence of LIF as previously described (26, 32). All cells were maintained at 37 °C in a humidified incubator with 5% CO_2_.

### *In vitro* synthesis of RNA

Synthetic RNA were prepared by *in vitro* transcription according to the methods that have been previously described (18). Briefly, the DNA template for enhanced GFP mRNA (GFP-mRNA) was generated from a pEGFP-N1 plasmid (BD Biosciences) by PCR using Q5 High-Fidelity DNA polymerase (New England BioLabs). The resulting dsDNA templates contain the T7 φ2.5 promoter for *in vitro* transcription (49, 50), the 5′-UTR region with a Kozak sequence (51), and the open reading frame of GFP. To prepare functional GFP, *in vitro* transcription from the DNA templates was carried out in the presence of the cap analog m^7^GpppA (chemically synthesized in our lab, unpublished) to generate 5′-capped GFP-mRNA transcripts. The purified RNA transcripts were polyadenylated by *E. coli* Poly(A) polymerase (New England Biolabs), resulting in functional mRNA, m^7^GpppA-GFP-polyA (designated as GFP-mRNA).

To prepare double-stranded RNA (dsRNA), dsRNA containing 650 nucleotides that nearly encompass the entire length of GFP-mRNA and dsRNA containing 606 nucleotides of Gausia luciferase mRNA (simplified as dsGFP and dsLuc, respectively) were prepared by annealing the sense and antisense RNA transcribed from separate templates of the same sequences but with a T7 φ2.5 promoter in opposite directions, therefore having a perfect dsRNA structure. B2RNA is a 178 nucleotide RNA sequence corresponding to the B2 SINE that is abundantly distributed in the mouse genome (30). It can fold into a secondary structure with five hairpins, two loops, and two single stranded regions (31). B2RNA was amplified by PCR from both mouse genomic DNA and cDNA with the primer pair 5′-GGGCTGGTGAGATG-3′ and 5′-AAAGATTTATTTATTTATTATA-3′. The DNA fragment was cloned into a pB2 plasmid downstream of a T7 promoter by our previously developed *in vivo* cloning method (52). Restriction digestion by BsaI at the end of the B2 sequence resulted in a linearized DNA molecule, from which B2RNA was synthesized by transcription with T7 RNA polymerase. Cy3-labeled B2RNA (Cy3-B2) was prepared similarly but in the presence of synthetic Cy3-AMP that acts as a transcription initiator to yield fluorescent RNA (53)

### Cell transfection and treatment

ESCs and D−/−ESCs were plated at 40 to 50% confluence and were usually cultured for 24 h before the experiments. Synthetic RNA were transfected into the cells with Endofectin Max (Genecopoeia) at 300 ng/ml for GFP-mRNA, B2RNA, and polyIC (Sigma-Aldrich) and 200 ng/ml for dsGFP and dsLuc, or at the concentrations specified in individual experiments. The imidazolo-oxindole PKR inhibitor (C16, Sigma-Aldrich) was used to inhibit PKR activity as previously described (25). To determine the effects of secreted IFNβ on ESCs and D−/−ESCs transfected with RNA, the cells were incubated with IFNβ-neutralizing antibodies or isotype control antibodies (BioLegend) for 30 min prior to RNA transfection. The cellular responses to type I IFN were determined with mouse recombinant IFNα (eBioscience). The treated cells were collected and used for various analyses under conditions described in individual experiments.

### Preparation of conditioned medium (CM) and ELISA analysis of IFNβ

CM preparation was carried out with protocols previously described (54). Briefly, ESCs and D−/−ESCs were transfected with dsGFP (200 ng/ml). After 4 h, the medium was removed, and cells were thoroughly washed with PBS. The cells were then cultured in DMEM that contains 15% FBS for an additional 24 h. CM prepared from cells without transfection with dsGFP was used as control CM. The CM was collected and centrifuged at 10,621*g* for 15 min, and the supernatants were collected and used for the treatment of ESC-FBs. To analyze IFNβ secreted to the culture medium, ESCs and D−/−ESCs were transfected with dsGFP (1 μg/ml) in serum free medium with 0.2% BSA. After 24 h, the culture medium was collected, concentrated with a centrifugal concentrator (10 kd cutoff), and analyzed for IFNβ protein using a quantitative mouse ELISA kit (BioLegend) according to the manufacturer’s instruction.

### Microscopy and flow cytometry analysis of GFP expression

Cell morphology and viability were routinely monitored with an Olympus CKx31 phase-contrast microscope during the time course of treatment. Expression of GFP in live cells was visualized using a Leica fluorescence microscope (DFC3000G). The images were acquired with a digital camera mounted on the microscope. The cell population expressing GFP was quantitatively determined by flow cytometry with an Accuri C6 flow cytometer (BD Biosciences) (18). Cell gating was performed by selecting the cell population from forward *versus* side scatter (FSC *versus* SSC) dot plots to exclude debris. Histograms that represent GFP expression levels were generated from median fluorescence intensities of analyzed samples with CFlow software (BD Biosciences) as previously described (18).

### Cell viability and cell cycle analysis

The viability of ESCs and D−/−ESCs was determined by cell number and by cell morphology after toluidine blue (TB) staining. The absorbance at 630 nm of TB-stained cells was measured with a microtiter plate reader. The values, which correlate with the number of viable cells, were used as an indirect measurement of cell proliferation or viability. Cell cycle analysis was performed with an LSRFortessa flow cytometer (BD Biosciences) after the cells were stained with 50 μg/ml propidium iodide. The cell cycle profiles were generated using FlowJo software.

### siRNA transfection and PKR knockdown

siRNA targeting PKR and negative control siRNA (Santa Cruz Biotechnology) were transfected to the cells with Endofectin Max at a final concentration of 100 nM. The cells were then analyzed for siRNA knockdown efficiency and the effect on cell proliferation under the specified experimental conditions.

### Western blot analysis

Protein analysis by Western blot was performed according to our published method (25). The antibodies against β-Actin, STAT1, PKR, pPKR, p19, p21, CDC25A, and CDK2 were purchased from Santa Cruz Biotechnology and peIF2α antibodies were from Cell Signaling Technology.

### Real-time quantitative polymerase chain reaction (RT-qPCR)

Total RNA was extracted using Tri-reagent (Sigma). cDNA was prepared by MMLV reverse transcriptase (Promega). RT-qPCR was performed using SYBR green ready mix on an MX3000PTM RT-PCR system (Stratagene), as previously reported (48). The mRNA level from RT-qPCR was calculated using the comparative Ct method (55). β-Actin mRNA was used to normalize relative levels of mRNA for tested genes. The sequences of the primer sets are listed in Table 1.

**Table 1:** The primer sequences used for RT-qPCR

Gene	Forward primer (5′-3′)	Reverse primer (5′-3′)
β-Actin	CATGTACGTAGCCATCCAGGC	CTCTTTGATGTCACGCACGAT
PKR	AAGCAGGAGGCAAGAAACG	TGACAATCCACCTTGTTTTCGT
RIG-I	ATTCAGGAAGAGCCAGAGTGTC	GTCTTCAATGATGTGCTGCAC
MDA5	CGATCCGAATGATTGATGCA	AGTTGGTCATTGCAACTGCT
IFNβ	CCCTATGGAGATGACGGAGA	ACCCAGTGCTGGAGAAATTG
ISG15	AGGTCTTTCTGACGCAGACTG	GGGGCTTTAGGCCATACTCC
B2 RNA	GAGTTCAAATCCCAGCAACCA	TACACTGTAGCTGTCTTCAGACA
IFNAR1	GACAACTACACCCTAAAGTGGAG	GCTCTGACACGAAACTGTGTTTT
IFNAR2	TGTCTGCGAGCCTAGAGACTA	AGCCGGGAATTTCGTATTGTTAT
JAK1	ACGCTCCGAACCGAATCATC	GTGCCAGTTGGTAAAGTAGAACC
TyK2	TGCATCCACATCGCACACAA	CTCCTGGGGATTCATGCCA
STAT1	GCTGCCTATGATGTCTCGTTT	TGCTTTTCCGTATGTTGTGCT
STAT2	CTGAAGGACGAACAGGATGTC	CAGGGTGGTTAATCGGCCAA
p16	CGCAGGTTCTTGGTCACTGT	TGTTCACGAAAGCCAGAGCG
p19	ATGCTGGATTGCAGAGCAGTA	ACGGGGCACATTATTTTTAGTCT
p21	CGAGAACGGTGGAACTTTGAC	CAGGGCTCAGGTAGACCTTG
Cyclin E1	CCTCCAAAGTTGCACCAGTTTGC	GACACACTTCTCTATGTCGCACC
Cyclin A2	CAGTCACAGGACAGAGCTGG	GGGCATGTTGTGGCGCTTTG
Cyclin B1	CGAGAACTGCTCTTGGAGACATTG	CCTGACACAGATACTCTTCTGCAG

### Statistical analysis

Statistical analysis was performed with Microsoft Excel using a two-tailed and unpaired Student’s *t*-test. Data are presented as the mean ± SD under specified experimental conditions. Statistical differences are indicated by *p*-values. *p* < 0.05∗ was considered statistically significant. *p* values up to four significant digits are shown as specified in figure legends.

## Data availability

All data described in this study are either presented in the main article or in the supporting information.

## Conflict of interest

The authors declare that they have no conflicts of interest with the contents of this article.
