# Supplementary material for: Dicer represses the interferon response and the double-stranded RNA-activated protein kinase pathway in mouse embryonic stem cells
Source: J Biol Chem. 2021 Jan 8;296:100264. doi: 10.1016/j.jbc.2021.100264 (PMC7948645; doi:10.1016/j.jbc.2021.100264)
Supplement: Supplemental Figures S1 and S2 [file mmc1.pdf]

*Supporting Information*

# Dicer acts as a repressor of interferon response and the double-stranded RNA-activated protein kinase pathway in mouse embryonic stem cells

Chandan Gurung[1], Mona Fendereski[1], Krishna Sapkota[2], Jason Guo[1†], Faqing Huang[2], and Yan-Lin Guo [1*]

[*] **Corresponding author:** Yan-Lin Guo, Ph.D.
E-mail: yanlin.guo@usm.edu

**Supporting material included:** Figure S1 and Figure S2

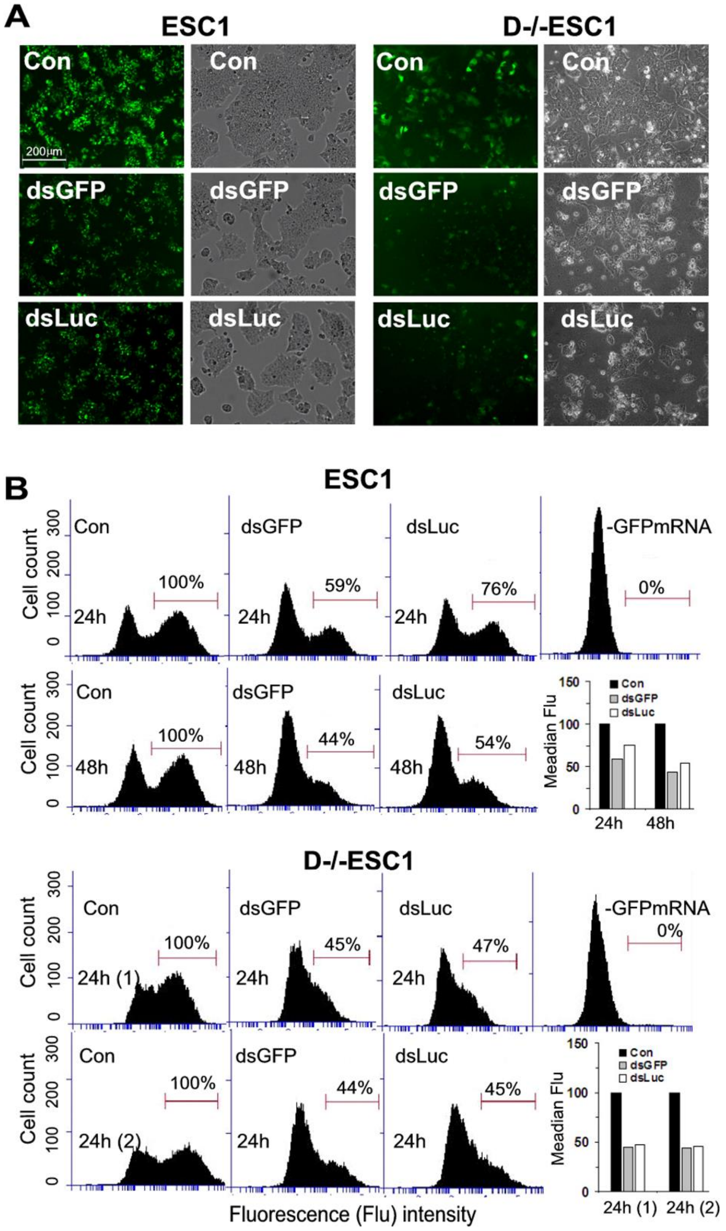


**Figure S1. Determining RNAi activity using GFP as a target in ESC1 and D-/-ESC1**. Cells were first transfected with GFP-mRNA. After 3-5 h, the cells were then transfected with dsGFP or dsLuc. Cells transfected with GFP-mRNA only were used as the control (Con). The cells were cultured for an additional 24 h or 48 h. GFP expression and the effects of dsGFP and dsLuc were analyzed by microscopy (**A**, 24 h) or by flow cytometry (**B**, 24h and 48 h). Fluorescnce (Flu) intensity represents the relative levels of GFP expression. The number of GFP positive cells in the control (Con) was set to 100%. Cells without any transfection were set to 0% as the baseline. The bar graphs show the median GFP Flu intensities of tested samples shown in the histograms. For D-/-ESC1, the data were from cells of two independent experiments that were cultured for 24 h after transfection with dsGFP and dsLuc. D-/-ESC1 that were cultured for 48 h could not be analyzed due to strong cytotoxicity caused by RNA transfection. All experiments were repeated at least twice with similar results.

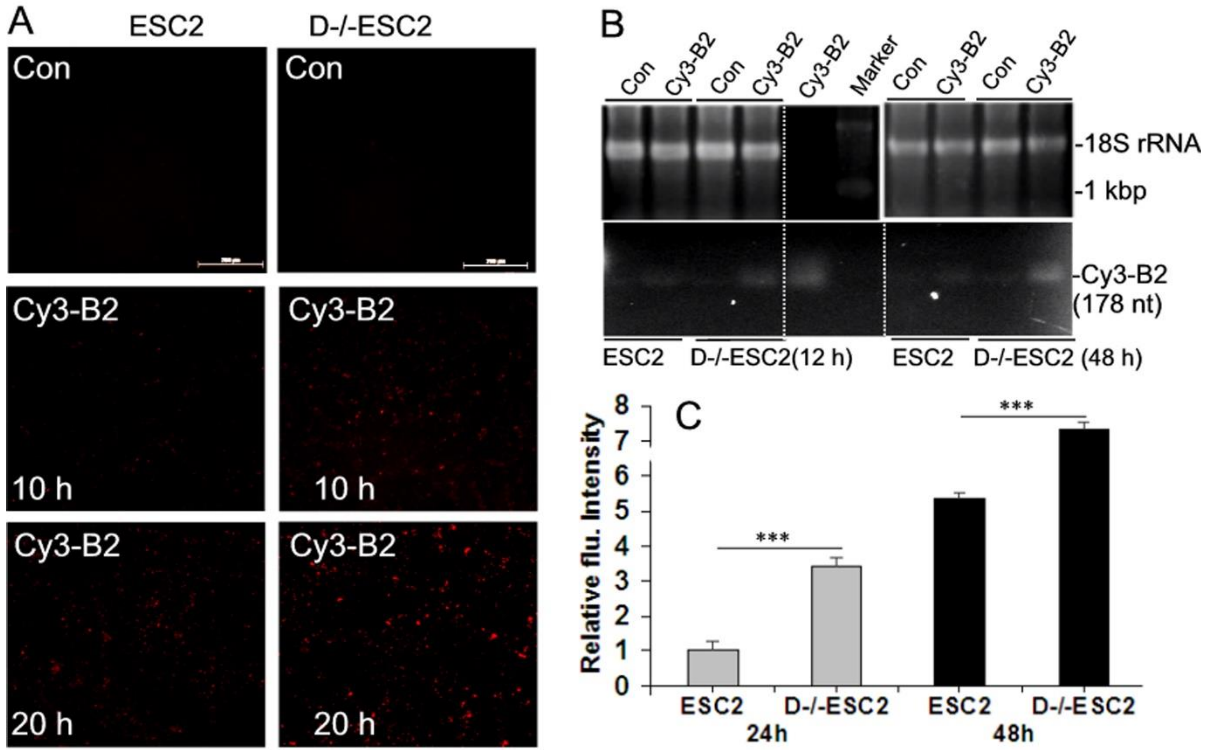


**Figure S2. Reduced degradation of B2RNA in D-/-ESC2**. **A,** ESC2 and D-/-ESC2 were transfected with 400 ng/ml of Cy3-labelled B2RNA (Cy3-B2) and examined under a fluorescence microscope at indicated times. Con represents the cells without Cy3-B2 transfection. The images are from a representative experiment that was repeated twice with similar results. **B,** Total RNA (3μg) extracted from the treated cells was analyzed by agarose gel electrophoresis. Cy3-B2 was identified by Cy3-fluorescence, and 18S rRNA was stained with ethidium bromide. The images were obtained by a phosphorimager (Bio-Rad, Molecular Imager FX). **C,** The relative amount of Cy3-B2 in ESC2 and D-/-ESC2 shown in B was quantified by the fluorescence intensity of Cy3-B2 normalized to that of 18S rRNA using ImageJ software. The value in ESC2 was defined as 1. The values are as mean ± SD of a representative experiments performed in biological triplicate that were performed at least twice. $P<0.0001$,****; $P<0.001$,***; $P<0.01$,**; $P<0.05$.* Compared groups are indicated by a horizontal bar.
